# The Buschke–Ollendorff syndrome: a case report of simultaneous osteo-cutaneous malformations in the hand

**DOI:** 10.1186/s13104-016-2095-2

**Published:** 2016-06-07

**Authors:** Michael Brodbeck, Q. Yousif, P. A. Diener, M. Zweier, J. Gruenert

**Affiliations:** Department of Hand Plastic and Reconstructive Surgery, Cantonal Hospital, St. Gallen, Switzerland; Department of Pathology, Cantonal Hospital, St. Gallen, Switzerland; Institute of Medical Genetics, University of Zurich, Schlieren-Zurich, Switzerland

**Keywords:** Osteopoikilosis, Dermatofibrosis lenticularis disseminata, Buschke–Ollendorff syndrome, LEMD3

## Abstract

**Background:**

We describe a male with functionally impairing radial deviation of the thumb who presented to us at 24 years of age. Two sclerotic skin lesions had been excised 7 years before because of consecutive skin contracture. Latest radiological examination showed a spotted pattern consistent with osteopoikilosis.

**Case presentation:**

A corrective osteotomy of the thumb was carried out due to the patients discomfort. Facing the simultaneous osteo-cutaneous malformation we postulated a Buschke–Ollendorff syndrome. Buschke–Ollendorff syndrome is a rare autosomal-dominant hereditary disorder of connective tissue with typical osteo-cutaneous manifestations. To explore our hypothesis, biopsies were taken from the affected bone lesions and surrounding skin and soft tissue for histological investigation and genetic testing of the *LEMD3* gene was performed on blood of the patient. The histology showed typical changes of the bone architecture and a fibrotic collagenous nodule of the skin. The genetic testing on DNA extracted from peripheral blood leucocytes confirmed a heterozygous loss of function mutation in the LEM domain-containing protein 3 (*LEMD3*) gene coding for the inner nuclear membrane protein MAN1, which causes osteopoikilosis by antagonizing transforming growth factor β (TGF-β) and bone morphogenetic protein (BMP) signalling.

**Conclusions:**

In atypical cases of simultaneous occurrence of fibrotic skin lesions and a spotted pattern in the X-ray we recommend the genetic screening of the *LEMD3* gene. A correct diagnosis of Buschke–Ollendorff syndrome is necessary to spare patients from expensive investigations and to provide reassurance about the benign nature of the disease.

## Background

Buschke–Ollendorff syndrome (BOS) is a rare hereditary disorder of connective tissue. It is inherited in an autosomal-dominant pattern with high penetrance. Multiple cutaneous elastic hamartomas and osteopoikilosis are the two key features for Buschke–Ollendorff syndrome. Two skin lesion patterns have been described as they may be of either elastic tissue (juvenile elastoma) or collagenous composition (dermatofibrosis lenticularis disseminata) [1]. The approximate incidence of the disease is 1:20,000, with few cases reported in the literature since 1928 [2, 3]. Skeletal lesions known as osteopoikilosis are areas of increased bone density that can be seen on radiographic imaging and typically are located in the substantia spongiosa of the epiphyses and metaphyses of long bones and the pelvis as well as in the hand.

## Case report

A 24-year-old male presented to our clinic with a history of painless and slowly progressive radial abduction of his right thumb affecting grip and pretension. He complained increasingly discomfort while working at the computer. No history of specific trauma or any other symptoms have been reported. Seven years earlier two sclerotic skin lesions over the radial aspect of the distal third of the first metacarpal bone have been excised.

**Fig. 1:**
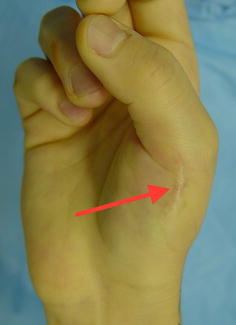
Radial angulation of the thumb and hypertrophic scar of the soft tissue nodule excision 7 years before. The *arrow* marks the area where the skin and soft tissue biopsy was taken

On physical examination, the patient looked healthy with no dysmorphic features. No skin lesions were detected. Only his right thumb was radially deviated 60° in the metacarpophalangeal (MP) joint and 25° in the interphalangeal (IP) joint (Fig. 1). There was a scar of previous operation over the radial aspect of the base of the thumb. The patient showed full active range of motion and the thumb joints were stable.

**Fig. 2:**
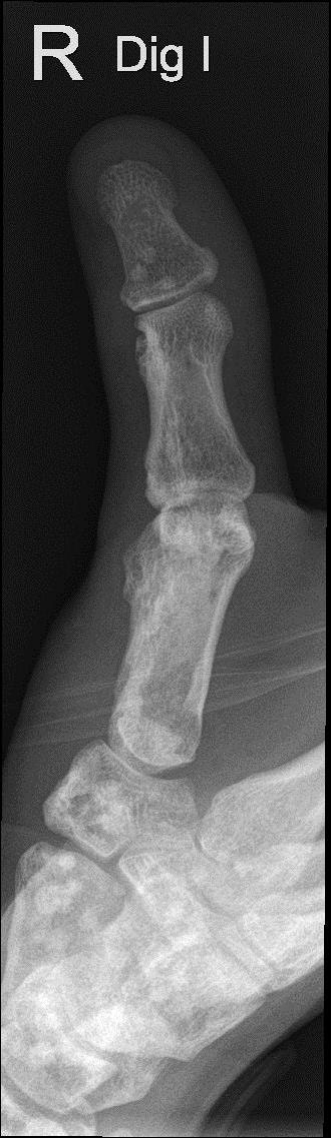
Osteopoikilosis (“spotty bone”)

Radiological examination of the hand showed a spotted pattern with multiple circular or ovoid sclerotic lesions, consistent with osteopoikilosis. Growth disturbance during childhood led to radial deviation in the IP and MP joints (Fig. 2). Before presentation to our unit the association of bony and soft tissue lesions was not correlated to a well-defined syndrome.

**Fig. 3:**
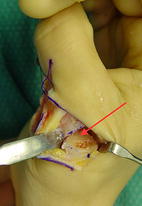
Medial closing wedge osteotomy of the proximal phalanx. Note the thickened sclerotic cortical bone

Due to the patients discomfort treatment with corrective osteotomy was carried out and a fixation plate with bicortical screws for early functional rehabilitation was mounted. Intraoperatively the cortical bone appeared highly sclerotic and thickened (Fig. 3).

**Fig. 4:**
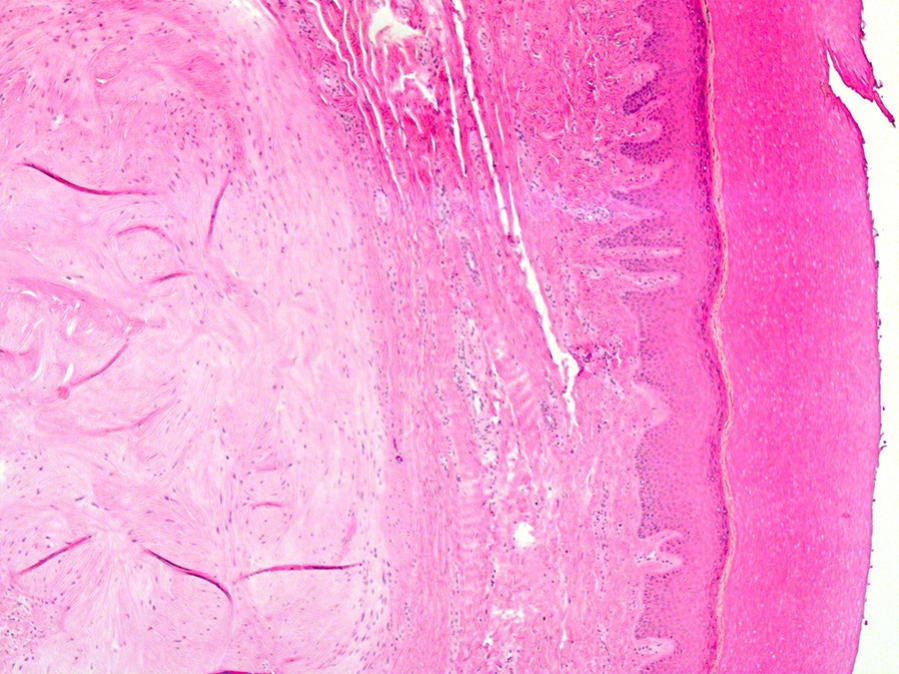
Histological findings in the skin (H&E, 50×): normal epidermis. Intradermal hypocellular collagenous nodule

**Fig. 5:**
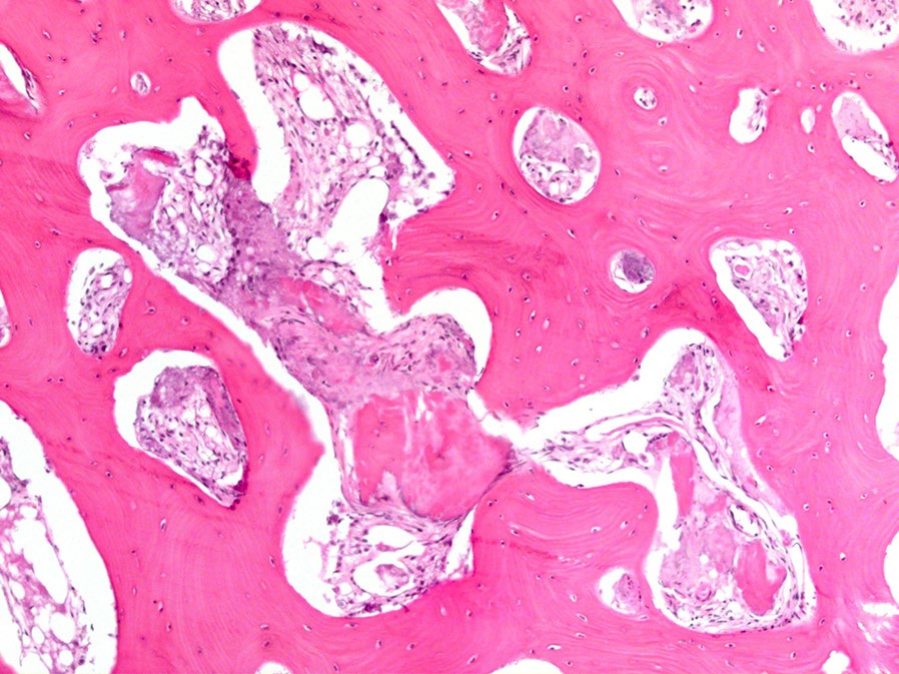
Histological findings in the bone (H&E, 200×): area of thickened cortical bone with remodelling and formation of numerous enlarged Havesian canals

To further investigate the possibility of BOS in the patient biopsies were taken from the affected bone lesions and surrounding skin and soft tissue over the former surgical scar (Fig. 1). The skin biopsy showed macroscopically a white nodule in the dermis. Microscopically, the nodule was hypocellular and consisted of interlacing thick collagen fibres and rare elastic fibres (Fig. 4). A segmental biopsy of the metacarpal bone of the right thumb showed a nodular thickening of the cortical bone. Histology revealed in the nodular thickening of the cortical bone a remodelling with formation of network of thick bone trabecules and formation of numerous enlarged Havers’s channels with fibrotic tissue (Fig. 5). The histological findings of the bone were consistent with osteopoikilosis and the skin showed the variant of a collagenous fibrotic nodule instead of a nodule with high concentration of elastic fibres.

**Fig. 6:**
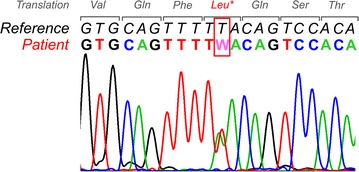
Electropherogram from Sanger sequencing confirming the heterozygous mutation c.1832T>A detected by next generation sequencing in our patient. The mutation in exon 6 of the *LEMD3* gene is predicted to result in a premature stop codon at position 611 (p.Leu611*) of the protein (reference sequence *LEMD3* according to GenBank NM_014319.4)

To confirm the diagnosis of BOS we obtained informed consent from the patient, isolated DNA from peripheral blood leucocytes and analysed the entire coding sequence of *LEMD3* with next generation sequencing (NGS) using the Agilent SureSelect XT Clinical Research Exome Kit (V5) on a HiSeq 2500 System (Illumina Inc.). This revealed the heterozygous mutation c.1832T>A, which was confirmed by Sanger sequencing and is predicted to result in a premature stop codon (p.Leu611*), probably leading to an incomplete gene function (Fig. 6, reference sequence GenBank NM_014319.4). To our knowledge, this mutation is not yet described in the literature or in the Human Gene Mutation Database (HGMD^®^) [4].

## Discussion

In BOS, cutaneous lesions consist of elastin or collagen naevi. Phenotypic expression of the disease is variable, and skeletal and cutaneous lesions may occur separately [5]. Cultured fibroblasts of patients with BOS produce 2–8 times more tropoelastin than fibroblasts of healthy individuals. Elastin production is higher in involved and uninvolved skin [6–9]. Elevated elastin mRNA levels suggest that BOS may result from abnormal regulation of extracellular matrix, leading to increased levels of elastin mRNA and increased accumulation of elastin in the dermis.

Osteopoikilosis is caused by heterozygous loss of function mutations in the LEM domain-containing protein 3 46 (*LEMD3*) gene, also known as *MAN1* [7]. LEMD3 is an inner nuclear membrane protein that antagonizes transforming growth factor β (TGF-β) and bone morphogenetic protein (BMP) signalling [10, 11]. BMP and TGF-β regulate bone turnover and skin development.

A *LEMD3* investigation in a two-generation BOS family showed extreme intrafamilial clinical variability of *LEMD3* mutations, which underlines the lack of a clear phenotype–genotype correlation in BOS [12]. Another case describing the absence of *LEMD3* mutation in an affected family may indicate the genetic heterogeneity of Buschke–Ollendorff syndrome [13]. Thus far, more than 22 mutations have been identified in the *LEMD3* gene [14].

The otosclerosis with hearing impairment, stenosis of the aorta, and diabetes found in this syndrome can lead to serious conditions even if these symptoms are rare [9, 10, 15]. However, in general BOS follows a benign course. The associated lesions are generally asymptomatic, begin in childhood, and persist throughout life, often found as incidental findings [16].

The history of skin thickening causing discomfort to the patient in the thumb signifies the presence of dermal induration caused by connective tissue nodules. A similar case was described by Kobus et al. in a six-year-old male with BOS [17]. The skin biopsy of our patient showed macroscopically a white nodule in the dermis. Microscopically, the nodule was hypocellular and consisted of interlacing thick collagen fibres and rare elastic fibres. These findings are not typical for BOS, which normally shows higher elastin production in involved and uninvolved skin [6–8]. However, phenotypic expression of BOS is variable, and skeletal and cutaneous lesions may occur separately [3]. The genetic testing identified a novel loss of function mutation in the *LEMD3* gene, which confirmed the diagnosis of BOS.

After corrective osteotomy of the thumb a normal postoperative course followed with osseous consolidation in time. Overall the patient stated a benefit of our treatment, which led to better function in his activity of daily living.

## Conclusions

Genetic screening of the *LEMD3* gene is advisable in case of atypical presentation or previous surgical treatment. A correct diagnosis of Buschke–Ollendorff syndrome is necessary to spare patients from expensive investigations and to provide reassurance about the benign nature of the disease. Osteopoikilosis can look very worrisome in X-rays of patients prior to diagnosis of BOS. These radiologic findings can lead to a lot of unnecessary work-up and anxiety in patients.

## Abbreviations

BMP: bone morphogenetic protein
BOS: Buschke–Ollendorff syndrome
DNA: deoxyribonucleic acid
IP: interphalangeal
HGMD^®^: human gene mutation database
LEMD3: LEM domain-containing protein 3
MAN1: inner nuclear membrane protein
MP: metacarpophalangeal
TGF-β: transforming growth factor β
